# Genetic Control of Tolerance to Drought Stress in Wild Soybean (*Glycine soja*) at the Vegetative and the Germination Stages

**DOI:** 10.3390/plants13141894

**Published:** 2024-07-09

**Authors:** Thi Cuc Nguyen, Hai Anh Tran, Jeong-Dong Lee, Hak Soo Seo, Hyun Jo, Jong Tae Song

**Affiliations:** 1 Department of Applied Biosciences, Kyungpook National University, Daegu 41566, Republic of Korea; nguyenthicuc.cttk57@gmail.com (T.C.N.); haianhctt57@gmail.com (H.A.T.); jdlee@knu.ac.kr (J.-D.L.); 2 Department of Agriculture, Forestry and Bioresources, Seoul National University, Seoul 08826, Republic of Korea; seohs@snu.ac.kr

**Keywords:** abiotic stress, drought stress, wild soybean, next-generation sequencing, association mapping

## Abstract

Drought stress, which is becoming more prevalent due to climate change, is a significant abiotic factor that adversely impacts crop production and yield stability. Cultivated soybean (*Glycine max*), a versatile crop for humans and animals, exhibits sensitivity to drought, resulting in reduced growth and development under drought conditions. However, few genetic studies have assessed wild soybean’s (*Glycine soja*) response to drought stress. In this work, we conducted a genome-wide association study (GWAS) and analysis of wild soybean accessions to identify loci responsible for drought tolerance at the vegetative (*n* = 187) and the germination stages (*n* = 135) using the available resequencing data. The GWAS analysis of the leaf wilting score (LWS) identified eight single-nucleotide polymorphisms (SNPs) on chromosomes 10, 11, and 19. Of these, wild soybeans with both SNPs on chromosomes 10 (adenine) and 11 (thymine) produced lower LWS, indicating that these SNPs have an important role in the genetic effect on LWS for drought tolerance at the vegetative stage. At the germination stage, nine SNPs associated with five phenotypic measurements were identified on chromosomes 6, 9, 10, 13, 16, and 17, and the genomic regions identified at the germination stage were different from those identified for the LWS, supporting our previous finding that there may not be a robust correlation between the genes influencing phenotypes at the germination and vegetative stages. This research will benefit marker-assisted breeding programs aimed at enhancing drought tolerance in soybeans.

## 1. Introduction

Soybean [*Glycine max* (L.) Merr.] is primarily cultivated as a source of protein for animal feed and vegetable oil for human use. It is among the most important commercially farmed legume crops worldwide [1]. In addition to amino acids, dietary minerals, vitamins, and nutraceuticals such as isoflavones and tocopherols, it is an inexpensive source of high-quality protein (40% of its dry mass) and edible oil (20% of its dry mass) [2]. It is estimated that around 12,000 foods include soy protein, and the diversity of soy products is still expanding [2,3].

Although numerous experts have studied drought tolerance, ongoing research into drought stress remains essential because enhancing drought tolerance is a critical objective in crop breeding amidst increasing global warming and climate change. Initially, drought stress can drastically alter the physiological and anatomical characteristics of the plant [4]. Drought, for example, has been shown to decrease the relative water content of roots and fresh root weight [5], the levels of chlorophyll [6], stomatal conductance [7], photosynthetic efficacy, and biomass [8] and increase canopy temperature [9], yet many other factors are still not well understood. Despite some small genetic contributions, most genes controlling the complex trait of drought tolerance are essential for genetically increasing drought tolerance [10]. Soybean is a drought-sensitive crop [4,11], and genetic improvement of drought tolerance is an important strategy for maintaining yield during drought conditions. Drought-resistant features must be incorporated into soybean’s genetic resources to develop varieties that preserve sustainable crop yields [12].

Slow wilting is linked to moisture conservation. Fast-wilting genotypes exhaust soil moisture reserves relatively quickly [13,14], while for slow-wilting genotypes, the conservation of soil water appears to be linked to decreased hydraulic conductance under high vapor pressure deficit, which reduces transpiration and improves water-use efficiency [15,16]. The quantitative trait loci (QTLs) mapping of drought-related traits has been carried out in soybean, with particular attention to yields under drought stress conditions [17,18], fibrous roots [19], water-use efficiency [20,21], and canopy wilting [22,23,24,25]. According to several studies, canopy wilting is a complicated trait mainly influenced by QTLs or interactions between QTLs and environmental factors [17,18,22,23,24,25,26,27]. Research into the QTLs’ underlying drought tolerance during the germination stage is scarce. Thus, breeders find it challenging to use genetic information for drought stress at germination stages since the molecular mechanism underlying soybeans’ drought resistance during the germination stage is unknown [28].

Genome-wide association studies (GWAS) enable the identification of genomic regions associated with specific traits by utilizing diverse soybean germplasm, especially that of cultivated soybean germplasm. Several studies in *G. max* soybean have used the GWAS approach for different traits under drought conditions, such as canopy wilting [25,26,28,29], germination rate [28,30,31,32], various nitrogen traits [33], canopy temperature [34], and carbon 13 ratio plasticity [35]. However, little genetic information is available for its ancestor, wild soybean (*Glycine soja* Sieb and Zucc.), regarding its response to drought stress at either the vegetative or germination stages. Thus, this study aimed to identify the genomic regions responsible for drought-related traits at the vegetative and germination stages in wild soybean through GWAS analyses based on single-nucleotide polymorphisms (SNPs) from available resequencing data.

## 2. Results

### 2.1. Phenotypic Distribution

**Figure 1:**
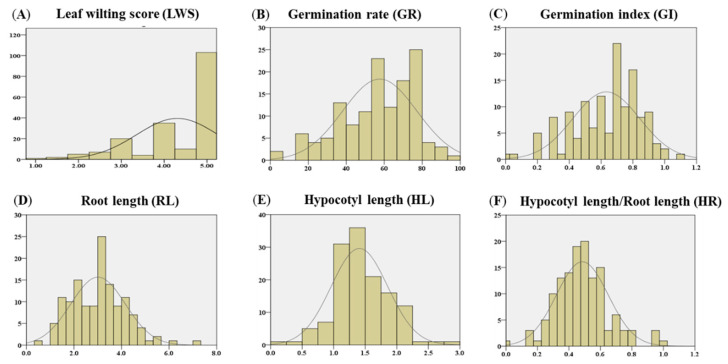
Phenotypic distributions of leaf wilting score at the vegetative stage and phenotypic measurements at the germination stages for wild soybean accessions under drought stress. (**A**) Leaf wilting score (LWS). (**B**) Germination rate (GR). (**C**) Germination index (GI). (**D**) Root length (RL). (**E**) Hypocotyl length (HL). (**F**) The ratio of hypocotyl length to root length (HR).

The leaf wilting score (LWS) was recorded under drought stress to investigate the phenotypic variation in seedling growth responses to drought. The LWS was significantly affected by drought stress. The frequency distribution of 187 soybean accessions for LWS is illustrated in Figure 1. Based on our previous study’s results [36], highly tolerant genotypes showed less than 1.5 of LWS values, tolerant accessions showed LWS values between 1.5 and 2.5, moderate genotypes had LWS values between 2.5 and 3.5, sensitive genotypes showed LWS values between 3.5 and 4.5, and highly sensitive accessions showed greater than 4.5 of LWS values. The result showed that one accession was highly tolerant, and seven were tolerant to drought stress. The proportions of moderate, sensitive, and highly sensitive were 12.83% (24 accessions), 18.72% (35 accessions), and 60.43% (113 accessions), respectively. The analysis of variance (ANOVA) results for the LWS trait is shown in Table 1. Significant differences in the LWS were found among accessions (*p* < 0.0001).

The frequency distributions of 135 soybean accessions for the germination rate (GR), germination index (GI), root length (RL), hypocotyl length (HL), and the ratio of hypocotyl length to root length (HR) are depicted in Figure 1. The mean values of GR, GI, RL, HL, and HR were 57.6, 0.6, 3.0, 1.4, and 0.5, respectively. ANOVA showed a significant effect of accessions (*p* < 0.0001) for all five traits (Table 1). Correlation analysis showed that GR was strongly correlated with GI (*r* = 0.956, *p* < 0.01) (Table 2), while other drought-related traits at the germination stage were either weakly correlated or not significantly correlated. The results showed that GR was positively correlated with RL (*r* = 0.280, *p* < 0.01) and SL (*r* = 0.245, *p* < 0.01) but not correlated with HR (*r* = 0.024, not significant). Similarly, GI was positively correlated with RL (*r* = 0.257, *p* < 0.01) and SL (*r* = 0.236, *p* < 0.01) but not correlated with HR (*r* = 0.045, not significant). However, RL was negatively correlated with HR (*r* = −0.403, *p* < 0.01) and positively correlated with HL (*r* = 0.450, *p* < 0.01). Additionally, HL was positively correlated with HR (*r* = 0.438, *p* < 0.01).

### 2.2. GWAS Results

#### 2.2.1. SNPs Associated with Drought Tolerance in Wild Soybean at the Vegetative Stage

**Figure 2:**
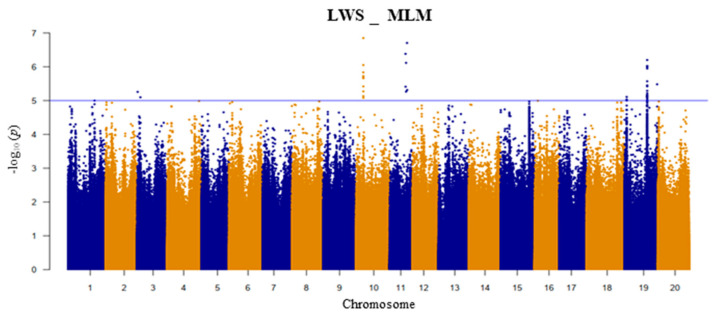
Manhattan plot for the leaf wilting score (LWS) using mixed linear model (MLM) method. The *x*-axis represents the chromosomes; the *y*-axis represents the −log10 (*p*) values. The blue line indicates the suggestive threshold.

This study used a diverse set of 187 *G. soja* accessions. After excluding 20% of missing SNP data and SNPs with minor allele frequencies (MAFs) ≥ 5%, we obtained 8,775,931 SNPs for further analyses. The GWAS was analyzed based on a mixed linear model (MLM) of the LWS of plants at the vegetative stage (Figure 2). The summarized results of the GWAS analysis and SNPs with −log_10_ (*p*) values ≥ 5.0 for the LWS trait are presented in Table S1. We identified that SNPs for the LWS trait at the vegetative stage were detected on chromosomes 3, 10, 11, and 19.

However, in a GWAS analysis based on the fixed and random model circulating probability unification (FarmCPU) method, eight significant SNPs were located on chromosomes 10, 11, and 19 (Table 3, Figure S1). These loci were used to find the variation among accessions at the eight positions where allele variation occurred. All showed significant associated changes in LWS, as determined using *t*-tests (Table 3). The interactions of these SNPs are presented in Table 4. Based on the reference soybean genome (Wm82.a2.v1), SNPs D10_11361356 on chromosome 10, D11_26601868 on chromosome 11, and D19_34790292 on chromosome 19 have the reference nucleotides adenine (A), guanine (G), and cytosine (C), whereas the alternative nucleotides were thymine (T), A, and G, respectively. The change from G to A on chromosome 11 more significantly affects LWS than the SNPs on chromosomes 10 and 19, and its appearance always causes the LWS to be less than or equal to 3 (Table 4). The interaction between the SNPs on chromosomes 10 and 11 produces a lower average LWS than the other SNP interactions (Table 4).

#### 2.2.2. SNPs Associated with Drought Tolerance in Wild Soybean at the Germination Stage

**Figure 3:**
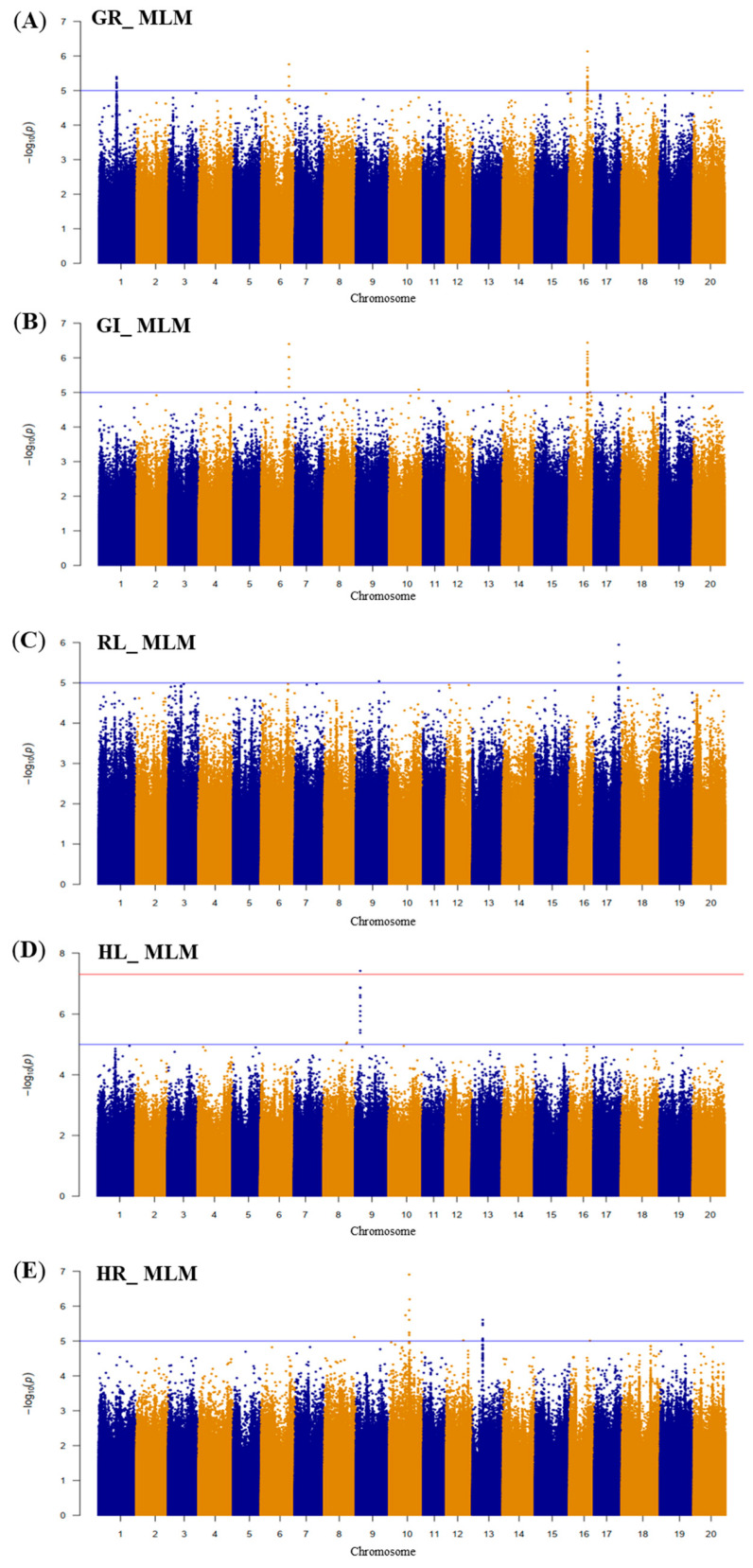
Manhattan plots of the SNPs tested for phenotypic measurements at germination stages using a mixed linear model (MLM). (**A**) Germination rate (GR). (**B**) Germination index (GI). (**C**) Root length (RL). (**D**) Hypocotyl length (HL). (**E**) The ratio of hypocotyl length to root length (HR). The *x*-axis represents chromosome; *y*-axis represents the −log10 (*p*) values. The blue line indicates the suggestive threshold, and the red line indicates the significance threshold.

First, the GWAS analysis was conducted using the MLM method for the phenotypic measurements at the germination stage (Figure 3). The summarized results and the SNPs with −log10 (*p*) values ≥ 5.0 for the GR, GI, RL, HL, and HR traits are presented in Table S2. The GWAS analysis revealed that SNPs associated with GR are located on chromosomes 1, 6, and 16; SNPs associated with GI are located on chromosomes 5, 6, 10, 14, and 16; SNPs associated with RL are located on chromosomes 9 and 17; SNPs associated with HL are located on chromosomes 8 and 9; and SNPs associated with HR are located on chromosomes 8, 10, and 13.

The nine most significant SNPs, based on a FarmCPU analysis with a Bonferroni-corrected threshold, are listed in Table 5 and Figure S2. These SNPs were used to find the variation among accessions at the positions where allele variation occurred, assessing significance using *t*-tests. Among the nine, one allele variation associated with GI did not show a significant effect, while the other eight significantly affected the associated trait (Table 5). One overlapping SNP on chromosome 16 (D16_28071218) was associated with both GR and GI.

### 2.3. Putative Genes Associated with the Significant SNPs for Drought Tolerance

#### 2.3.1. Putative Genes Associated with the Significant SNPs for LWS

We examined the soybean reference genome Wm82.a2.v1 within a 20 kbp range of the most significant SNPs to identify potential candidate genes. Table 6 summarizes the 22 candidate genes with relevant annotations retrieved from Soybase (http://www.soybase.org, accessed on 1 February 2024). Six of these genes are associated with LWS at the vegetative stage. These are annotated in the public database as, respectively, being involved in the alpha/beta hydrolase fold, the hAT family C-terminal dimerization region, copper/zinc superoxide dismutase (SODC), the helix–loop–helix DNA-binding domain, the zinc finger C_3_HC_4_ type (RING finger)/CHY zinc finger, and the B3 domain-containing transcription factor fus3.

#### 2.3.2. Candidate Genes Associated with the Significant SNPs for Germination-Stage Drought-Related Traits

We also examined the reference genome within a 20 kbp range of the most significant SNPs to identify potential candidate genes. Table 6 summarizes the 16 candidate genes and their relevant annotations. Two genes related to GR and GI were identified on chromosome 16, including a protein kinase domain and a 2OG-Fe (II) oxygenase superfamily gene. Another candidate gene was identified on chromosome 6 (a plastocyanin-like domain) and chromosome 16 (a GRAS domain family gene), which are associated with GI and GR, respectively. Two genes associated with RL were identified on chromosome 17, respectively annotated as “Zn-finger in ubiquitin-hydrolases and other protein” and “BT1 family”. Additionally, two genes related to HL were identified on chromosome 9; one was annotated as a “plant invertase/pectin methylesterase inhibitor”, and the other was identified as a gene of unknown function. Genes related to HR were identified on chromosomes 10 and 13 and annotated as “K+ potassium transporter/DNA polymerase alpha/epsilon subunit B”, “microtubule-associated protein”, “homeobox-leucine zipper protein”, “acyltransferase”, “LSM domain”, “UDP-glucoronosyl and UDP-glucosyl transferase”, “WD domain”, and “G-beta repeat” (Table 6).

## 3. Discussion

Soybean, a globally significant crop, faces considerable yield reductions due to drought stress. Developing drought-tolerant cultivars is crucial, with wild soybeans as valuable genetic resources. The wilting index is a practical tool to assess plant responses to drought stress on large scales [37,38]. This study utilizes wild soybean accessions from the Republic of Korea, China, Japan, and Russia [39], ensuring diverse genetic backgrounds suitable for GWAS aimed at pinpointing genomic regions linked to drought tolerance. The present study evaluated the drought response of 187 and 135 wild soybean accessions at the vegetative and germination stages, respectively. Eight wild soybean accessions with LWS values < 2.5 are suitable for use in breeding programs to develop drought-tolerant cultivars (Table S3).

Several different populations of cultivated soybeans were used to identify the chromosome regions associated with drought tolerance traits. Hwang et al. [25] identified nine QTL clusters associated with slow wilting located on chromosomes 2, 5, 11, 14, 17, and 19, and two meta-QTLs on chromosomes 11 and 19 were identified as major QTLs. However, the individual QTLs within these clusters were not consistently stable across different years [24]. Kwon et al. [40] identified a QTL region on chromosome 10 (*qSW_Gm10*) associated with a limited transpiration rate and sensitivity to the aquaporin inhibitor silver nitrate (AgNO3) that partially overlaps with a previously reported QTL [41]. This stable QTL (*qSW_Gm10*) interacts with a novel locus on chromosome 1 (*qSW_Gm01*). The combined effect of their alleles exceeded the sum of their individual additive effects, resulting in improved phenotypic values for wilting score and leaf moisture content. According to Chamarthi et al. [42], significant SNPs on chromosome 10 and chromosome 11 were consistently identified for drought tolerance across different environments. Another significant SNP associated with canopy wilting was identified on chromosome 10 [29]. In this study, significant SNPs on chromosomes 10 and 11 had an important genetic effect on leaf wilting at the vegetative stage (Table 4). Little GWAS research has been carried out using wild soybean accessions for their LWS under drought conditions. Therefore, this study’s findings enhance our knowledge of the genetic mechanisms governing drought tolerance in wild soybeans during the vegetative stage.

We used the SoyBase database to identify potential genes linked to significant SNPs. A total of 22 annotated genes were found within 20 kbp of the significant SNPs associated with all tested drought-related traits (Table 6). Many of these genes encode proteins involved in plant stress responses, such as the hAT family C-terminal dimerization region, copper/zinc superoxide dismutase, the helix–loop–helix DNA-binding domain, and the zinc finger protein. In soybean, gene expression data have demonstrated the involvement of *GmCCS7*/*GmCCS24* (encode copper chaperone for superoxide dismutase) in the control of drought tolerance. Increased SOD and other antioxidant enzyme activities were observed in soybean hairy roots expressing *GmCCS7/GmCCS24*, indicating better resistance to drought stress [43]. Elevated levels of dehydroascorbate reductase and chloroplastic superoxide dismutase [Cu-Zn] may offer antioxidant-related defenses against drought damage in rice [44,45] and sweet potato [46]. A potential gene associated with LWS was found to be the homologous gene *Glyma.11g192700*, which is annotated “copper/zinc superoxide dismutase (SODC)” and located in the same region as a QTL discovered by Hwang et al. [25], *mqCanopy wilt-009*. Thus, *Glyma.11g192700* may be related to drought tolerance at the vegetative stage in this study. Further research is needed to validate the putative drought tolerance genes identified in this study by conducting linkage analyses and comparing the expression levels of these genes in drought-treated and controlled soybean plants.

Seed germination is an important stage in overall crop growth and, ultimately, crop production. The soybean GR under drought conditions is a quantitative trait, and several studies have examined the drought responses of soybean at the germination stage [28,30,31,32]. Liu et al. [28] reported eleven SNPs that showed significant associations with the GR on chromosomes 5, 6, 11, 12, 13, 14, 17, 18, 19, and 20 and three SNPs significantly associated with RL on chromosomes 9, 17, and 20 from the GWAS analysis on 259 cultivated soybeans. Zhao et al. [30] conducted a GWAS analysis on 410 soybean accessions and identified eight SNPs related to the relative GR on chromosomes 1, 4, 5, 8, 11, and 20. Another study identified 28 SNPs that were significantly linked to GR in two different environments on chromosomes 3, 4, and 18 [31]. In addition, Sun et al. [32] identified five SNPs on chromosomes 1, 2, 6, 10, and 20 that were associated with a germination index and one SNP on chromosome 10 that was associated with a main RL index. Thus, based on GWAS studies looking at soybeans at the germination stage under drought conditions, QTLs associated with GR and RL phenotypes are influenced by different genetic backgrounds of an association panel. In addition, there are no reported GWAS analyses of wild soybean accessions examining drought tolerance at the germination stage. In this study, five phenotypic index values showed significant variation among 135 wild soybean accessions (Figure 1). Our GWAS analysis identified nine significant SNPs related to drought tolerance in wild soybean at the germination stage: two associated with GR on chromosome 16; two and one associated with GI on chromosomes 6 and 16, respectively; one associated with RL on chromosome 17; one associated with HL on chromosome 9; and two associated with HR, one each on chromosomes 10 and 13 (Table 5). These identified genomic regions differ from those of previously reported GWAS studies of cultivated soybean [28,30,31,32]. In addition, the genetic controls of GR under drought conditions were different from those of RL based on GWAS results at the germination stages of this study.

The 2-OGD gene (*Pn2-ODD1*) was discovered in *Pohlia nutans* by Wang et al. [47]. Overexpression of this gene enhanced the plants’ ability to cope with salinity and drought stress in Arabidopsis and *Physcomitrella patens*. Abiotic stress responses may be significantly influenced by 2-OGDs, as demonstrated by Chelliah et al. [48]. The gene that encodes the 2OG-Fe (II) oxygenase superfamily, *Glyma.16g128700*, was identified as a potential candidate for GR and GI traits. However, the genetic mechanisms associated with RL and HL under drought stress were different from those associated with the GR and GI in this study. A C_2_H_2_ zinc finger protein assisted plants in responding to abiotic stress by increasing abscisic acid (ABA), proline, carbohydrates, and chlorophyll or decreasing the rate of water loss [49], and the soybean zinc finger protein gene *GmRZFP1* may be involved in signal pathways associated with responses to drought, high salt, high temperature, low temperature, ethylene, and ABA stressors [50]. In this study, *Glyma.17g218300* (Zn finger in ubiquitin hydrolases and other proteins) was found to be a potential gene influencing RL under drought conditions in soybeans. Additional research is needed to validate the roles of identified genes in drought tolerance at either vegetative or germination stages. Comparisons of gene expression levels between drought-stressed and control soybean plants will be crucial in confirming their involvement. These findings are expected to provide deeper insights into the regulatory mechanisms governing drought responses in soybeans.

Our previous study indicated that drought-related assessments at the vegetative and reproductive stages did not seem to correlate with the GR and RL at the germination stage under drought conditions [36]. For example, wild soybean accessions that were drought tolerant at the vegetative and reproductive stages showed significantly lower GRs and RLs at the germination stage under drought conditions than under the control condition. Similarly, in barley [51], there was no correlation between drought tolerance assessments at the germination and vegetative stages. In this study, the genomic regions associated with phenotypic measurements at the germination stage were different from those associated with the LWS, corroborating our previous study’s findings [36] (Table 4 and Table 5). Drought tolerance is a complex trait governed by multiple genetic loci. Understanding the genetic basis of drought tolerance in wild soybeans at various growth stages holds potential for bolstering resilience in cultivated soybean varieties. To confirm the genomic regions from the GWAS result, linkage analysis will be required using biparental mapping populations, which can be developed from the drought-tolerant wild soybean accessions in this study.

In conclusion, this study evaluated 187 and 135 *G. soja* accessions for drought tolerance at the vegetative and germination stages, respectively. ANOVA identified significant differences among the genotypes in drought-related traits, including LWS, GR, GI, RL, HL, and HR. A GWAS analysis was performed using 8,775,931 SNPs. Eight and nine significant SNPs related to drought tolerance at the vegetative and germination stages, respectively, were detected. Wild soybeans with SNPs on chromosomes 10 and 11 produced a lower leaf wilting score than other allele combinations. Thus, these SNPs were considered to play an important role in the genetic effect on leaf wilting at the vegetative stage. In addition, the genomic regions associated with phenotypic measurements at the germination stages were different from the ones associated with the LWS, supporting the findings of our previous study that there may not be a robust correlation between the genes influencing measured phenotypes at the germination and vegetative stages. The identification of SNPs associated with the GR, GI, RL, and HL in this study indicated that a different genetic basis was involved in the drought stress responses of RL and HL than was involved in the GR and GI. These findings will be useful for marker-assisted breeding programs aimed at enhancing drought tolerance in soybeans.

## 4. Materials and Methods

### 4.1. Plant Materials

This study utilized a diverse panel of wild soybean accessions sourced from the National Agrobiodiversity Center of the Rural Development Administration in Jeonju, Republic of Korea (https://genebank.rda.go.kr/, accessed on 1 February 2024) (Table S3). One hundred eighty-seven wild soybean accessions were selected to assess phenotypic responses to drought stress at the vegetative stage, and one hundred thirty-five wild soybean accessions were selected to evaluate phenotypic responses to drought stress at the germination stage.

### 4.2. Phenotypic Evaluations

#### 4.2.1. Leaf Wilting Scores (LWSs) of Wild Soybean at the Vegetative Stage

A phenotype analysis of drought stress-treated accessions was conducted under glasshouse conditions at Kyungpook National University, Daegu, Republic of Korea (36°06′45.8″ N 128°38′33.4″ E). The LWS was measured using the plastic tray method described by Nguyen et al. [36]. In the experiment, five seeds were initially planted in each hole of plastic trays (46 × 23 × 11 cm) filled with horticultural soil (Hanareum; Shinsung Mineral, Goesan, Republic of Korea). The seedlings were then thinned to two plants per hole, with each hole representing a single replication. The experiment was conducted in duplicate under controlled conditions with a 14 h light and 10 h dark cycle. Soybean plants at the V2 stage, characterized by two trifoliate leaves, were subjected to drought conditions for seven days, and drought tolerance was assessed using LWS for each accession. The LWS ranged from 1 to 5, where 1 indicates no wilting, 2 indicates 1–25% wilting, 3 indicates 26–50% wilting, 4 indicates 51–75% wilting, and 5 indicates that the entire plant was dead. Three repeated experiments were conducted (29 August to 21 September 2023, 1 September to 25 September 2023, and 4 September to 30 September 2023), and an average of the LWSs was used for the GWAS analysis.

#### 4.2.2. Drought-Related Traits in Wild Soybean at the Germination Stage

A total of 135 *G. soja* accessions developed by the Rural Development Administration, Jeonju, Republic of Korea, were used to evaluate the phenotypic response to drought tolerance at the germination stage [52]. The drought conditions were generated by treating germinating seeds with 12% PEG 6000, as described by Nguyen et al. [36]. In this experiment, ten healthy seeds from each accession were placed on wet filter paper within 9 cm diameter Petri dishes. These Petri dishes were filled with either 10 mL of PEG 6000 solution or distilled water (as a control). Seeds with roots at least 1 cm long were considered germinated. From each accession, the five seeds with the longest roots were selected, and their root lengths (RL) and hypocotyl lengths (HL) were measured. The ratio of HL to RL, known as HR, was also recorded. The germination experiment was repeated three times to determine the overall germination percentage. The germination rate (GR) and germination index (GI) were calculated using the following equations:

GR = (number of germinated seed)/(number of sowed seed) × 100

GI = GRd/GRn

where GRd and GRn represent the GR under drought (PEG 12%) and control (distilled water) conditions, respectively.

### 4.3. GWAS Analysis

In order to identify the genetic loci controlling drought tolerance at the vegetative and germination stages, we gathered whole-genome sequence (WGS) data for the 187 and 135 accessions of wild soybean, respectively [53]. These sequencing data were mapped to the Wm82.a2.v1 reference genome of soybean Williams 82 [54]. The SNPs with MAFs under 5% were eliminated to remove low-quality SNPs.

A GWAS analysis was conducted to identify loci controlling drought response, followed by candidate gene identification. The MLM method was used to perform the association analysis. The MLM was applied to evaluate the dataset using the Genome Association and Prediction Integrated Tool (GAPIT) package in R [55]. Manhattan plots were drawn using the R package qqman [56]. Based on the MLM results, the FarmCPU method was used to separately analyze SNPs considering chromosomes in GAPIT. A threshold value of −log_10_ (*p*) incorporating the Bonferroni correction was adopted to identify significant associations between SNPs and phenotypic traits.

### 4.4. Putative Candidate Gene Identification

Significant SNPs were used to identify candidate genes putatively influencing drought-related traits using the *G. max* genome assembly version Wm82.a2.v1 (www.soybase.org, accessed on 1 February 2024) [54]. Genes located near the SNPs significantly associated with drought-related traits were considered potential candidates if they were within 20 kbp of the SNP. These distances were chosen to reflect the average distance between SNPs based on the linkage disequilibrium decay in wild soybean. Candidate genes were identified and categorized to be associated with drought tolerance-related responses.

### 4.5. Statistical Analysis

Data analysis was performed using SPSS (IBM SPSS Inc., Chicago, IL, USA). Analyses of variance (ANOVA) were conducted, and descriptive statistics, including means and standard deviations, were calculated for each trait. The phenotypic frequency distributions of the drought response traits were produced, and the degree of association between traits was analyzed based on Pearson’s correlations [57]. Statistically significant differences in trait values between SNP groups were assessed using *t*-tests.

## Figures and Tables

**Table 1:** Mean square values for the analyses of variance (ANOVA) of soybean drought-related traits.

Traits	Source of Variation	Degree of Freedom	Sum of Squares	Mean Sum of Squares	*F*-Value	*p*-Value
LWS	Accession	186	501.810	2.700	195.9	<0.0001
	Replication	2	0.040	0.020	1.6	0.206
GR	Accession	134	142,396.100	1062.657	1.4	<0.0001
	Replication	2	2359.717	1179.858	1.5	0.321
GI	Accession	134	11.916	0.089	0.7	<0.0001
	Replication	2	0.309	0.154	1.2	0.389
RL	Accession	134	351.931	2.646	34.7	<0.0001
	Replication	2	0.360	0.180	2.4	0.299
HL	Accession	134	53.692	0.407	16.0	<0.0001
	Replication	2	0.035	0.018	0.7	0.504
HR	Accession	134	7.041	0.053	15.8	<0.0001
	Replication	2	0.007	0.004	1.1	0.346

LWS, leaf wilting score; GR, germination rate; GI, germination index; RL, root length; HL, hypocotyl length; HR, ratio of hypocotyl length to root length.

**Table 2:** *r* values for the correlation analyses of drought-related traits at the germination stage.

	GR	GI	RL	HL	HR
GR	1				
GI	0.956 **	1			
RL	0.280 **	0.257 **	1		
HL	0.245 **	0.236 **	0.450 **	1	
HR	0.024	0.045	−0.403 **	0.438 **	1

GR, germination rate; GI, germination index; RL, root length; HL, hypocotyl length; HR, ratio of hypocotyl length to root length; **, statistically significant at *p* < 0.01.

**Table 3:** Most significant SNPs associated with the leaf wilting score (LWS), as identified using the FarmCPU method.

Trait	Chr	Physical Position	−log_10_(*p*)	Mean LWS Associated with the SNP Allele				*t*-Test	MAF	Allelic Effect
				A (n)	T (n)	C (n)	G (n)			
LWS	10	11,361,356	7.4	4.46 (136)	3.63 (35)			<0.0001	0.23	−0.47
	10	11,383,213	7.11		4.5 (129)	3.5 (22)		<0.0001	0.21	0.51
	11	26,601,868	26.26	2.71 (7)			4.38 (172)	<0.0001	0.06	1.04
	19	34,790,292	7.65			3.64 (42)	4.48 (134)	<0.0001	0.25	0.44
	19	34,790,013	7.39	3.69 (45)			4.47 (133)	<0.0001	0.26	0.42
	19	34,789,961	7.39	4.48 (131)	3.72 (46)			<0.0001	0.15	−0.78
	19	34,797,069	7.34	4.50 (129)	3.73 (44)			<0.0001	0.16	−0.74
	19	34,790,351	7.28	3.73 (44)			4.50 (129)	<0.0001	0.27	0.42

Chr, chromosome; MAF, minor allele frequency.

**Table 4:** Genotype variation of the most significant SNPs on chromosomes 10, 11, and 19 among 155 wild soybean accessions.

SNP	Genotypes							
	G1	G2	G3	G4	G5	G6	G7	G8 (Reference)
D10_11361356	T	T	A	A	T	T	A	A
D11_26601868	A	A	A	A	G	G	G	G
D19_34790292	G	C	G	C	C	G	G	C
Number of accessions	1	3	2	3	10	20	98	18
LWS ± SD	2.00 ± 0.00 ^nd^	2.00 ± 0.87 ***	2.50 ± 0.00 ***	2.33 ± 0.57 ***	3.42 ± 0.74 ***	3.80 ± 1.11 ***	4.72 ± 0.46 ^ns^	4.61 ± 0.50

A significance analysis was performed using G8 as the reference. Statistical significance was assessed using *t*-tests: ***, significant at *p* < 0.001; nd, not defined; ns, not significant (SD, standard deviation; LWS, leaf wilting score).

**Table 5:** Most significant SNPs associated with drought-related traits at the germination stage, as identified using the FarmCPU method.

Trait	Chr	Physical Position	−log_10_(*p*)	Mean Trait Score Associated with the SNP Allele				*t*-Test	MAF	Allelic Effect
				A (n)	T (n)	C (n)	G (n)			
GR	16	28,071,218	8.04	65.96 (76)			45.84 (37)	<0.0001	0.35	−10.60
	16	34,049,144	7.29		22.71 (7)	61.56 (123)		<0.0001	0.07	−19.53
GI	6	39,541,088	14.75		0.77 (124)	0.23 (6)		<0.0001	0.06	−0.19
	6	40,697,687	9.65		0.77 (13)	0.72 (95)		0.69	0.20	0.08
	16	28,071,218	7.99	0.86 (76)			0.46 (37)	<0.0001	0.35	−0.11
RL	17	36,893,010	7.42	2.93 (126)			5.33 (6)	<0.0001	0.05	1.30
HL	9	7,627,321	25.66	1.35 (124)			1.88 (8)	0.009	0.06	0.70
HR	10	30,512,307	11.81		0.39 (87)	1.00 (4)		<0.0001	0.49	−0.30
	13	15,434,946	6.99	0.37 (122)			0.78 (9)	0.025	0.08	0.14

Chr, chromosome; MAF, minor allele frequency; GR, germination rate; GI, germination index; RL, root length; HL, hypocotyl length; HR, ratio of hypocotyl length to root length.

**Table 6:** Putative candidate genes found within 20 kbp of the significant SNPs for each drought-related trait.

Trait	Chr	SNP	Gene	Start	End	Function (PFAM)
LWS	10	D10_11361356	*Glyma.10g087500*	11,350,383	11,356,511	Alpha/beta hydrolase fold
			*Glyma.10g087600*	11,364,689	11,366,137	hAT family C-terminal dimerization region
	11	D11_26601868	*Glyma.11g192700*	26,591,332	26,595,068	Copper/zinc superoxide dismutase (SODC)
			*Glyma.11g192800*	26,596,271	26,599,470	Helix–loop–helix DNA-binding domain
			*Glyma.11g192900*	26,604,592	26,608,357	Zinc finger, C_3_HC_4_ type (RING finger)
	19	D19_34797069	*Glyma.19g100900*	34,806,060	34,810,057	B3 DNA binding domain
GI	6	D06_39541088	*Glyma.06G239900*	39,530,793	39,532,182	Plastocyanin-like domain
GR, GI	16	D16_28071218	*Glyma.16g128600*	28,057,444	28,062,790	Protein kinase domain
			*Glyma.16g128700*	28,077,595	28,080,215	2OG-Fe (II) oxygenase superfamily
GR	16	D16_34049144	*Glyma.16g179900*	34,036,942	34,040,157	GRAS domain family
RL	17	D17_36893010	*Glyma.17g218300*	36,878,303	36,882,265	Zn-finger in ubiquitin-hydrolases and other protein
			*Glyma.17g218400*	36,900,654	36,906,204	BT1 family
HL	9	D09_7627321	*Glyma.09g072700*	7,619,132	7,620,402	Plant invertase/pectin methylesterase inhibitor
			*Glyma.09g072800*	7,633,793	7,634,182	Unknown
HR	10	D10_30512307	*Glyma.10g119600*	30,488,462	30,491,514	K^+^ potassium transporter/DNA polymerase alpha/epsilon subunit B
			*Glyma.10g119700*	30,491,587	30,494,187	Microtubule-associated protein (MAP65/ASE1 family)
			*Glyma.10g119800*	30,514,616	30,515,487	Homeobox-leucine zipper protein
			*Glyma.10g119900*	3,516,480	30,519,657	Acyltransferase
			*Glyma.10g120000*	30,522,225	30,525,686	LSM domain
	13	D13_15434946	*Glyma.13g056700*	15,423,364	15,424,333	Unknown
			*Glyma.13g056800*	15,431,070	15,433,820	UDP-glucoronosyl and UDP-glucosyl transferase
			*Glyma.13g05690*	15,439,351	15,446,633	WD domain, G-beta repeat

Chr, chromosome; LWS, leaf wilting score; GR, germination rate; GI, germination index; RL, root length; HL, hypocotyl length; HR, ratio of hypocotyl length to root length.

## Data Availability

The raw datasets used or analyzed during the current study are available from the corresponding author upon reasonable request.

## Supplementary Materials

The following supporting information can be downloaded at https://www.mdpi.com/article/10.3390/plants13141894/s1. Figure S1. Manhattan plots for the SNPs associated with the leaf wilting score (LWS), as determined using the FarmCPU method, on chromosome 10 (A), chromosome 11 (B), and chromosome 19 (C). The red line indicates the Bonferroni-corrected significance threshold. Figure S2. Manhattan plots for the SNPs associated with five drought-related traits during the germination stage, as determined using the FarmCPU method: (A) Manhattan plot for GR on chromosome 16, (B and C) Manhattan plots for GI on chromosome 6 (B) and on chromosome 16 (C), (D) Manhattan plot for RL on chromosome 17, (E) Manhattan plot for SL on chromosome 9, and (F and G) Manhattan plots for SR on chromosome 10 (F) and on chromosome 13 (G). The red line represents the Bonferroni-corrected significance threshold. Table S1: SNP loci associated with the leaf wilting score (LS) using the MLM model. Table S2: SNP loci associated with the drought-related traits at the germination stage using the MLM model. Table S3: List of the 243 accessions used in this study.
