# Supplementary material for: Genetic Control of Tolerance to Drought Stress in Wild Soybean (Glycine soja) at the Vegetative and the Germination Stages
Source: Plants (Basel). 2024 Jul 9;13(14):1894. doi: 10.3390/plants13141894 (PMC11281237; doi:10.3390/plants13141894)
Supplement: Supplementary file 1 [file plants-13-01894-s001.zip › Supplementary Materials_Figure_240606.pdf]

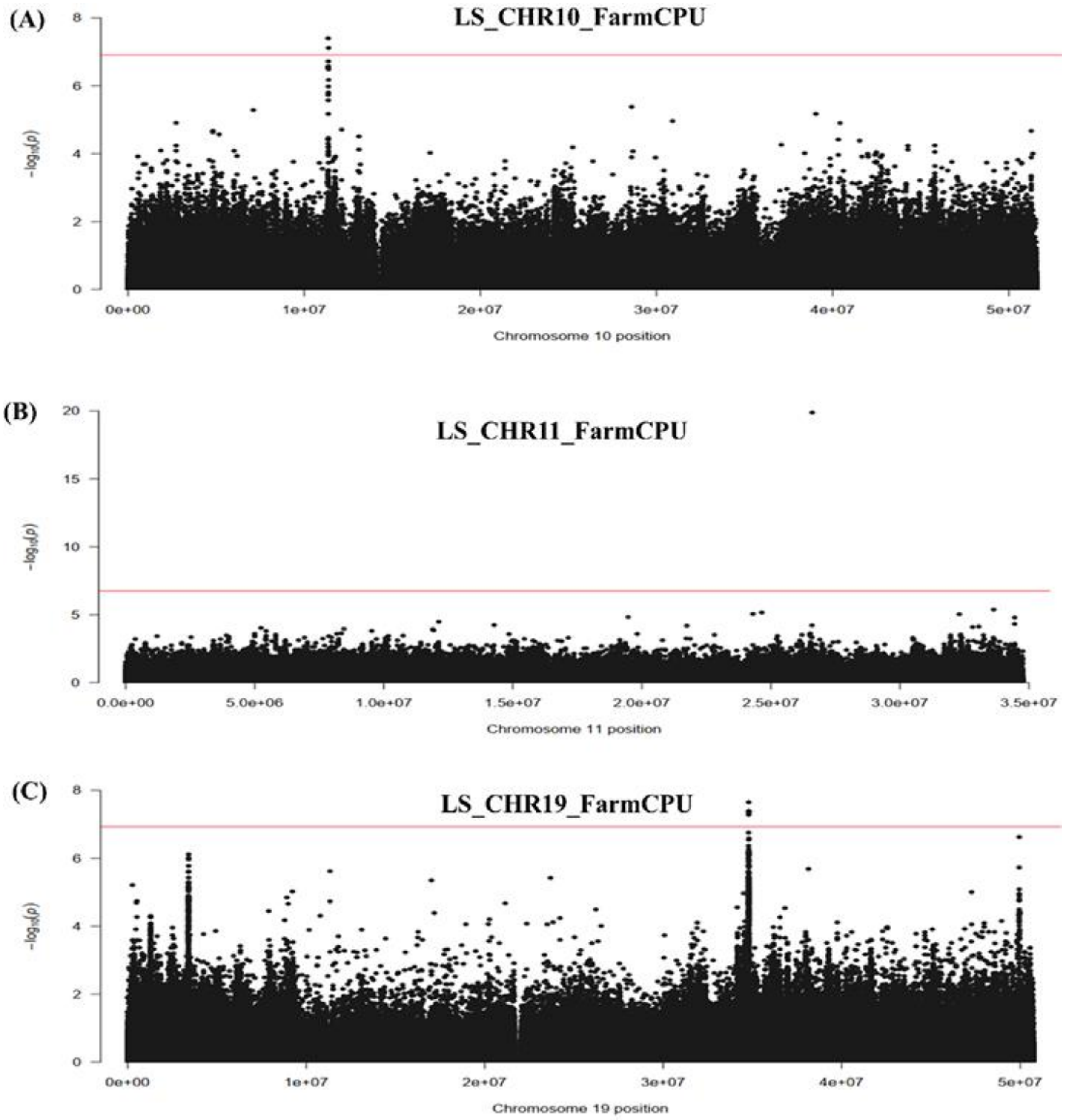

Figure S1. Manhattan plots for the SNPs associated with the leaf wilting score (LWS), as determined using the FarmCPU method, on chromosome 10 (A), chromosome 11 (B), and chromosome 19 (C). The red line indicates the Bonferroni-corrected significance threshold.

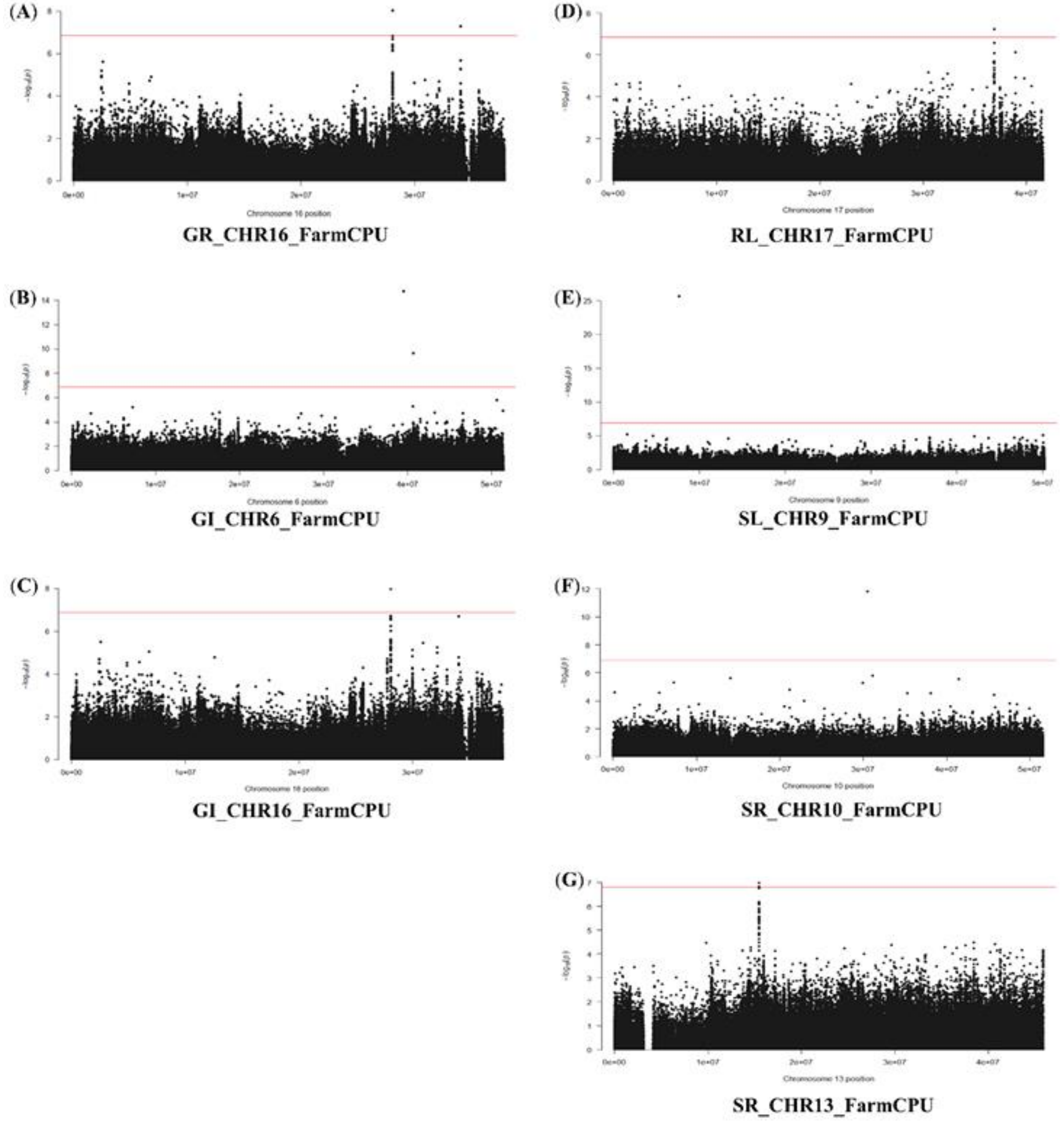

Figure S2. Manhattan plots for the SNPs associated with five drought-related traits during the germination stage, as determined using the FarmCPU method: (A) Manhattan plot for GR on chromosome 16, (B and C) Manhattan plots for GI on chromosome 6 (B) and on chromosome 16 (C), (D) Manhattan plot for RL on chromosome 17, (E) Manhattan plot for SL on chromosome 9, and (F and G) Manhattan plots for SR on chromosome 10 (F) and on chromosome 13 (G). The red line represents the Bonferroni-corrected significance threshold.
